# Children Prioritize Virtual Exotic Biodiversity over Local Biodiversity

**DOI:** 10.1371/journal.pone.0023152

**Published:** 2011-08-04

**Authors:** Jean-Marie Ballouard, François Brischoux, Xavier Bonnet

**Affiliations:** 1 Centre d'Etudes Biologiques de Chizé, CNRS-UPR 1934, Villiers en Bois, France; 2 Centre de Recherche et de Conservation des Chéloniens, SOPTOM, le Village des tortues, Gonfaron, France; 3 Department of Biology, University of Florida, Gainesville, Florida, United States of America; University of Pretoria, South Africa

## Abstract

Environmental education is essential to stem current dramatic biodiversity loss, and childhood is considered as the key period for developing awareness and positive attitudes toward nature. Children are strongly influenced by the media, notably the internet, about biodiversity and conservation issues. However, most media focus on a few iconic, appealing, and usually exotic species. In addition, virtual activities are replacing field experiences. This situation may curb children knowledge and concerns about local biodiversity. Focusing our analyses on local *versus* exotic species, we examined the level of knowledge and the level of diversity of the animals that French schoolchildren are willing to protect, and whether these perceptions are mainly guided by information available in the internet. For that, we collected and compared two complementary data sets: 1) a questionnaire was administered to schoolchildren to assess their knowledge and consideration to protect animals, 2) an internet content analysis (i.e. Google searching sessions using keywords) was performed to assess which animals are the most often represented. Our results suggest that the knowledge of children and their consideration to protect animal are mainly limited to internet contents, represented by a few exotic and charismatic species. The identification rate of local animals by schoolchildren was meager, suggesting a worrying disconnection from their local environment. Schoolchildren were more prone to protect “virtual” (unseen, exotic) rather than local animal species. Our results reinforce the message that environmental education must also focus on outdoor activities to develop conservation consciousness and concerns about local biodiversity.

## Introduction

Environmental education is one of the fundamental tools required to reverse the current trends in biodiversity loss [1]–[4]. Childhood is the key period to introduce environmental education owing to the strength and lasting quality of an early relationship formed between children and the natural world [5]–[9]. Using animals is particularly efficient in encouraging such a relationship, due to the affective relationship that children easily build with animals [10]. Animals, in general, may therefore provide an efficient means to connect people with their natural environment [10], [11]. In practice, personal experiences, knowledge and likeability are important determinants in the establishment of such a bond [12]–[16]. In addition, to develop positive attitudes towards global biodiversity, environmental education should encompass a wide diversity of species, notably by including less popular and neglected taxa [17]–[19]. Overall, environmental education programs should focus on children and should incorporate a broad range of species representative of global biodiversity.

Attitudes of children toward nature are influenced by family, personal experiences, media, and school [20]; with the prevalence of the media increasing over time. For instance, television occupies a central place in the lives of children [21]–[23], even supplanting the role of the family and substituting outdoor and social activities [24]. More recently, the internet has become the main source of information for children; it is also one of the main channels for social interactions. As a result, a strong shift in children's behavior with a considerable amount of time spent in front of a screen to the detriment of outdoor activities has been recently documented [25]–[29]. Importantly, current academic education systems favor the use of the internet. This form of media is indeed a major pedagogical tool for most teachers; for example, in 2005, almost 100% of public schools in the USA had access to the internet, compared with 35% in 1994 [30]. Internet access is considered as a major tool to connect children to the world, whilst field trips remain peripheral [30], [31]. As a consequence, the media (especially internet-based) are now the main channels providing information on species diversity and on environmental issues [32]. Accordingly, conservation educators rely on the internet to develop environmental consciousness and to raise concerns about biodiversity conservation [33].

In general, messages about conservation issues are based on a few iconic, flagship and “likeable” species (e.g., polar bear, dolphin, etc.) that benefit from a strong charismatic “cuddle factor” [17], [34], [35]. Therefore, the most demanded and easily accessible information on biodiversity is represented by exotic and appealing animals. This trend tends to “condition children to think that nature is exotic, awe-inspiring and in far, far away places, they will never experience” [36]. This situation likely explains the extremely poor level of knowledge of children about local biodiversity [18], [19] along with the detrimental disconnection between people and their biological environment [34], [37], [38].

Overall, children's everyday life has largely shifted to the indoors over the last decades [39]. Virtual information and vicarious experiences are progressively substituting direct and real personal experiences [34], [40], [41]. For instance, although children are able to recognize more than a thousand corporate logos, or hundreds of Pokémons along with their virtual life history traits [18], they can only identify a handful of animal and plants that are native to their home environment [18], [40]. In this context of growing virtualization we need to assess how these changes influence perception of local biodiversity, including knowledge and inclination to protect local environment [23], [42].

The research aims of the current study were to describe: 1) the level of French children's knowledge of animal biodiversity, focusing on the distinction between local and exotic species; 2) the situation in which local *versus* exotic species have been observed (e.g. in the field, on the internet…); 3) whether perceptions on which species should be protected differ with regard to locality of the species; 4) the level of diversity of the animals that are considered important to protect; and 5) the level of biodiversity presented on the internet. We explored several relationships between the 5 elements above. For instance, we evaluated the level of similarity between the animal biodiversity classified as deserving priority protection by children and the animal biodiversity obtained through internet content analysis. The outcome from such comparison should provide major clues to identify what guides conservation priorities declared by the children.

We used two techniques to collect the raw data. First, a questionnaire containing both open- and closed-ended items was administered to schoolchildren to assess their knowledge about local *versus* exotic animal species, and to examine which species they listed to protect in priority. Second, we performed an internet content analysis to assess which animals were the most often represented as threatened species. We performed our investigation in France. However, we extended the assessment of the children knowledge about local *versus* exotic animal biodiversity to a larger range of countries, including developed and developing nations, where the influences of different factors (e.g. social, cultural and level of education or local biodiversity availability) are contrasted. The study reported in this article forms part of a much larger, cross-national research project that aims to explore several issues in environmental education focusing on schoolchildren and animal biodiversity. We emphasize that the current study focused on a limited number of questions, especially to explore the potential influence (e.g., strong, or lack-of) of the internet on the declarations of schoolchildren with respect to a central question: which animal species should be protected in priority? The children provided a short list of the animals that must be protected; the selected species and their rank on the lists were used as a proxy for priority. We thereby did not attempt to examine the role(s) of other factors such as gender, age, environment, religion or culture for instance.

## Methods

As pointed out above, we employed two main data collection tools: a questionnaire-survey (i.e. a semi-structured questionnaire consisting of open- and closed-ended items) administrated to schoolchildren, and an image content searching performed on the internet by the authors.

### Semi-structured questionnaire administered to schoolchildren

Following preliminary tests [43], and after approval by a committee (see Ethics section), we administered a written questionnaire to schoolchildren (7–11 years old, 2007 and 2008) to assess their knowledge about animal biodiversity and their consideration to protect threatened species. This semi-structured questionnaire, consisting of open- and closed-ended items, was based on a total of 28 different main items (some contained multi-part questions that aimed to address both methodological and fundamental issues required for a large international project, not presented here). For the current study we used a subset of responses to the semi-structured questionnaire distributed during school time to 251 French schoolchildren from both rural and urban areas. The schoolchildren were drawn from 10 schools situated in the Middle-West of France. We sampled schools situated both in the country (N = 7, N = 164 schoolchildren) and in urban areas (N = 3, N = 87 schoolchildren), and the sex ratio was equilibrated (girls represented 52% of the total). All the schools had at least internet access, and all the children had also access to various media at home and/or through family and friend relationships. We ensured that the school classes were not previously involved in any educational program concerning animal biodiversity or wildlife threats. To limit the pressure on the schoolchildren, the questionnaire was introduced as a survey and not an exam. The observer (teacher) explained that the main goal was to assess the perception and knowledge about biodiversity in schoolchildren. The observer carefully avoided citing any precise example of threatened group of animals, and did not cite particular species (e.g., to introduce the questionnaire, the general term “animal” was used instead of “dolphin”). The observer also reminded the schoolchildren that organisms such as insects or worms belong to animals; otherwise many children would have overlooked invertebrates [44]. Then the observer distributed to each schoolchild a written semi-structured questionnaire consisting of open- and closed-ended items. Most of the children completed their individual questionnaire in less than 30 minutes. The children had to achieve two main tasks: responding to several open-ended questions and then to closed-ended questions by identifying various animals on a color plate.

#### Open-ended items

To assess which animal species schoolchildren spontaneously considered as deserving priority protection, we asked them to “list five animals that must be protected in priority”. The schoolchildren were also asked to explain where each of the cited species was observed: in the field, in their garden, in a zoo, on the television, in another media, etc. Animals were either really observed (i.e. living animal seen in the field or in a zoo for instance) *versus* virtually observed through a media (e.g. television, internet, magazine…). Several animals were observed in more than one situation (e.g. a fox can be seen in the field, in a zoo, or on television); others were almost exclusively observed in only one situation (e.g. giant panda in the media).

We analyzed the responses to gauge the diversity of the species that the children considered as essential to protect. We considered that the children understood the questions well (see below for further explanations) and that the list of five animals they provided largely reflected the species that they considered important to be protected in priority. Henceforth, for simplicity, we used the term “priority protection species”” (or “priority protection” when assessed as proportions) to refer to the species listed by the children. We retained in such a list of “priority protection species” all the animals cited by the children, irrespective of their actual conservation status (e.g. disregarding IUCN red list). Indeed, we aimed to poll the children, not to test if they correctly ranked animals in an official list of threatened species. This spontaneously listed biodiversity was then compared to the biodiversity presented in one of the most influential media: the internet (see Internet research design paragraph below).

#### Closed–ended items with color plates

Analyses of the data collected through schoolchildren surveys have shown that spontaneity is an important element that can influence children's answers and that can limit the number of local species listed (unpublished data). For instance, children tend to cite the species they recently observed. Consequently, domestic pets and exotic species were spontaneously over-cited as “priority protection species” (69.9%, unpublished data), somehow masking the biodiversity of local “priority protection species” that nonetheless potentially exists in the mind of the children.

To address this issue, we used an additional technique based on identification rate, rather than children's spontaneity. We provided each schoolchild with a color plate with twenty animals pictured in a standard way. We balanced the numbers of iconic exotic species (e.g., Polar Bear), non-iconic exotic species (e.g., Pangolin), iconic local species (e.g., Red Fox) and non-iconic local species (e.g., House Centipede). Importantly, none of the presented local species were cryptic (i.e., very difficult to observe); conversely we selected common and conspicuous animals easily spotted in gardens, city parks or at home (e.g., the European Black Bird, *Turdus merula*). We mixed species from six broad taxonomic groups (Mammals, Birds, Reptiles, Amphibians, Fish and Invertebrates). A total of 37 different species (N = 16 exotic species and N = 21 local species) were displayed on two different plates (3 species were identical on both plates). The two plates were presented to a total of 446 schoolchildren (N = 315 for the first plate and N = 131 for the second plate). For each picture, the children were asked if they had ever observed a live specimen (i.e., ever seen the animal in person regardless the location of the observation [in zoos, gardens, in the field, etc.]; but disregarding pictures, documentaries…), to provide precisely the name for each species (whenever possible, to the lowest taxonomic level), and then to list 5 species (among the 20 presented on the plate) that must be protected.

### Internet research design

Among various media (magazines, television, books, internet, etc.) we selected the internet for several reasons. Firstly, the internet is currently used by most school teachers as a predominant pedagogical tool. Secondly, it has been shown that the internet is also the prevalent media used by people to access scientific information [45], [46], and a schoolchild interested in a particular topic will use the internet as the most rapid, rich and convenient source of information. Thirdly, the prevalence of the internet is likely to increase over time, especially in the scholastic environment. Lastly, the similarities in the questionnaire and internet searching procedures allowed a straightforward comparison of the two datasets.

To produce a dataset comparable with the schoolchildren dataset, we used a realistic approach likely adopted by most children. Notably, we relied on the identification of animal species based on pictures obtained from the most used search tool (http://images.google.fr/) that can be defined as an internet picture content analysis. We used keyword-based searches using 6 different sets of keywords (i.e., “endangered animal”, “animal disappearing”, “animal extinction”, “protected animal”, “animal saved”, “threatened animal”) and duplicated this search by replacing “animal” by “species” (total of 12 different keyword phrases). Although this method likely oversimplified the current richness and complexity contained in various media outlets, and hence the potential impact on children's access to threatened animal information, we believe that it corresponds well to what young schoolchildren are experiencing during a comparable search session (pers. obs.). Indeed, virtually no child was aware of specialized websites (e.g., IUCN red list), and probably few would have been able to navigate or use them effectively.

For each set of keywords, we sampled twenty times 5 successive pictures (e.g., comparable to the five species listed by the schoolchildren, see above). Each picture was identified to the lowest taxonomic level (species level in most cases) by the three authors. We discarded duplicates (same picture associated to identical website). We obtained a total of 237 samples representing 1,185 animal pictures.

### Reliability of the data sets

Many possible sources of error (or observation effects) during data collection can influence the results, notably in the case of the open-ended questions (identification rate using color plates are less subjected to such bias). These include social desirability bias, especially considering the fact that children's perceptions were studied in the school setting, as well as schoolchildren's ability to reliably list and at the same time prioritize five species. In a parallel study (that forms part of the international project mentioned in the introduction) we assessed the reliability of the responses. We notably examined the ability of the schoolchildren to understand and accurately respond to several relatively complex and/or slightly different questions in order to perform cross-checking analyses (unpublished). Almost all the children (90%) correctly understood the goal of the study and accurately responded to the other questions. For instance, to the question “what are the causes of animal disappearance?” most of the answers (86%) correctly identified direct (e.g. poaching…) or indirect (e.g. habitat destruction…) factors, whilst only a few children provided out-of- focus (12%) or poorly formulated (2%) responses. We therefore estimated that the answers of the children were reliable.

The differing in level of biological knowledge between schoolchildren and the authors (who identified internet pictures) can influence the taxonomic level and the accuracy of the species identification (e.g., a “Humpback whale” would be accurately identified by the authors, but more likely classified as a “Whale” by most schoolchildren). This might artificially affect the similarity indices computed between samples (see below). As a consequence, we produced additional datasets adjusted to the taxonomic knowledge of schoolchildren (“top-down” approach). The images gathered from the internet were saved and re-identified by another group of schoolchildren (not involved in the other types of surveys) to the lowest taxonomic level (e.g., some bird species were simply named “bird”, but such imprecision applied equally to the entire data set, see below). In both datasets, species unknown to children but identified by the authors (e.g., the Aye-aye) for which re-nomination procedures would have been impossible to perform, were kept at the correct species level (e.g., the Aye-aye thus becomes the unknown species x).

### Statistical analyses

In order to compare the two datasets representing respectively the animal diversity of “priority protection species” perceived by the schoolchildren *versus* available on the internet, we used statistical approaches developed to compare the diversity patterns of different pseudo-communities, and we notably used estimates of species richness and shared species.

We performed richness estimates to test the effectiveness of our sampling of the diversity of “priority protection” species perceived by the schoolchildren and available on the internet (Chao estimator, [47]). We calculated similarity indices (Morisita-Horn index, [48], [49]) to quantify diversity overlap between schoolchildren and the internet. These analyses were performed using Estimates 8.2 [47]. Statistical estimates of species richness and shared species were performed on both raw and taxonomically adjusted datasets (see results). Other statistical analyses (contingency tables) were performed with Statistica 7.1.

### Ethics

The questionnaires we circulated to schoolchildren have been produced conjointly with (and approved by) schoolteachers and teachers specialized in child psychology [43]. The parents of the schoolchildren were all aware of our survey and our objectives which were clearly explained during meetings involving parents, teachers and ourselves. Because our survey was not considered as intrusive to schoolchildren by child psychologists and senior academy officers (i.e. “Inspecteur d'Académie” in France, all our investigations were performed in schools under the direct supervision of the “Education Nationale”) did not require any ethics approval. Similarly, Education Office (“Rectorat” in France) did not require any written consents from the parents of the schoolchildren.

## Results

### Sampling of “priority protection species”

**Figure 1:**
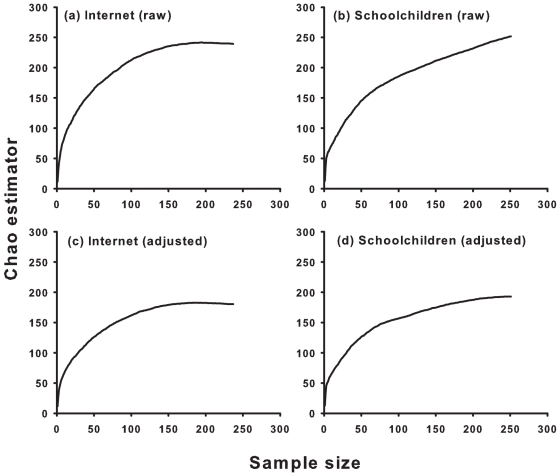
Sampling of “priority protection species”. The adequacy of sampling was based on richness estimator (Chao estimator) for the internet (raw and adjusted, N = 236 samples; see text for details) and schoolchildren (raw and adjusted, N = 250 samples; see text for details). All the curves reached a plateau, indicating that we adequately sampled the diversity of “priority protection species” both for the internet and for schoolchildren.

We collected approximately the same number of species both through the children's answers to the open questions (N = 166 species, from 1151 names cited, Fig. 1b) and with the internet (N = 184 species, from 1185 images, Fig. 1a). This was especially true when the taxonomic knowledge of the children was taken into account (taxonomically adjusted species names, see above; N = 144 species for the internet and N = 144 species cited by the children, Fig. 1c & 1d).

Overall, the richness estimator for both type of taxonomic precision plateaued after a sample size of ∼180 for the internet sample and ∼250 for the schoolchildren (Fig. 1), indicating that our sampling was adequate to quantify “priority protection species” diversity, as well as to compare the similarity of species diversity using both data collecting techniques ([47], see below).

### Similarity between the internet and schoolchildren

The diversity of “priority protection species” was broadly similar between the internet and schoolchildren samples. Focusing on the raw data (i.e., not taxonomically adjusted), over a list of 256 different species in total, 92 were in common between the two sources (children and the Internet). However, only few species were frequently cited and such over-cited species actually represented 80.5% of the samples. As a consequence, the computed Morisita-Horn similarity index was 0.663; indicating a broad similarity between the internet and schoolchildren samples [48], [49].

This similarity index was higher when the taxonomic knowledge of the schoolchildren was taken into account (taxonomically adjusted species names, see above): of the 202 “species”, 84 were in common between both samples, which represented 86.9% of the samples, leading to a relatively high Morisita-Horn index of 0.713 [48], [49].

### Perception of local versus exotic species

#### Observed species

**Figure 2:**
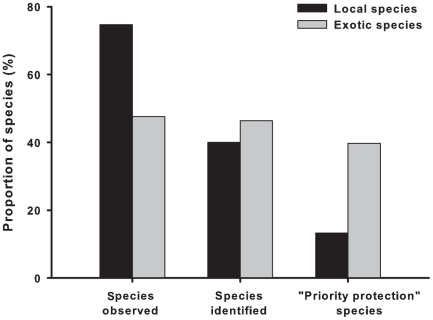
Local vs. exotic biodiversity. Proportion of exotic (grey bars) and local (black bars) species for which live specimens have been seen/observed by schoolchildren (“Species observed”), that were successfully identified by schoolchildren (“Species identified”) and that were perceived as “priority protection” by schoolchildren (“Priority protection species”). See text for details.

Overall, schoolchildren declared to have observed in person 61.1% of the species displayed on the color plates. As expected, local species were more often observed than exotic species (χ^2^ = 517.17, df = 1, p<0.0001; 74.7% of local species already observed *versus* 47.6% of exotic species, Fig. 2). A closer inspection of the data showed that the relatively high proportion of exotic species seen in person was explained by the fact that many cited species (e.g., elephants, lions) were observed in zoos. More precisely, such exotic animals were observed both virtually in the media, essentially television (53.8%), but also in person in zoos (50.8%). Only a very low proportion of children declared having already observed exotic species in their local environment (3.5%, possibly during a trip in a foreign country). We note that these results revealed a great level of honesty and understanding of the children, thereby strengthening the reliability of the findings.

#### Identification of pictured animals

Overall, schoolchildren were able to identify 43.1% of the species displayed on the pictures at a relatively precise taxonomic level (e.g., a “Bald Eagle” identified at least as an “Eagle” rather than as a “Bird”).

We detected a difference in the identification rates between the local and exotic species displayed on the pictures with the local species being less often identified than exotic ones (χ^2^ = 33.62, df = 1, p<0.001; 39.9% of local species *versus* 46.4% of exotic species identified to a correct taxonomic level, Fig. 2).

#### Animal considered essential to be protected

Overall, the mean “priority protection species” level (animals selected by the children) of the species displayed on the pictures was of 23.2% (range 2%–73.2% depending upon the species). Schoolchildren were more prone to protect exotic rather than local species (χ^2^ = 671.62, df = 1, p<0.0001; 39.7% of the species rated “priority protection species” were exotic, whereas only 13.3% were local species, Fig. 2).

Most of the “priority protection species” were highly iconic and exotic animals: the Giant Panda and the Polar Bear (respectively 73.2% and 71.1%). The less often identified species (the Green Rose Chafer, not recognized even at a broad taxonomic level) is a common and conspicuous (brightly colored) local insect species rated as “priority protection” solely by two schoolchildren.

## Discussion

We emphasize that our aim was not to investigate to what extent children were able to correctly cite or identify animals according to official classifications that are clearly intended for professional conservationists and managers (e.g. IUCN red list). Instead, we focused on the children's knowledge in relation to their declaration and willingness to protect certain animals; a key issue for a long-term perspective. Our results revealed strong and worrying bias: the diversity of species that should benefit from protection is meager, and more worrying, essentially guided by the narrow range of messages communicated by the media about very few iconic and usually exotic (mammalian) species. This clearly means that most of the biodiversity is neglected. This also suggests that one of the fundamental objectives of environmental education (e.g. officially declared by the French Education Nationale and by major international committees) is unsuccessful: most children may not be aware that protecting animal species at a local scale is of fundamental importance.

The generalization of our results to other countries could be a limitation to our conclusions. The spontaneous biodiversity of “priority protection species” we described in the current study might differ for children from other countries due to various social and biodiversity-related factors unique to France (or to Europe, or even western, developed countries) compared to developing nations. For instance, compared to the French children, those from other geographic areas may well have more comprehensive view of the biodiversity crisis. Unfortunately this is not the case. We performed similar surveys in Europe (Italia, Serbia, Slovakia, Spain, and Portugal, N = 1,107 schoolchildren involved), Africa (Morroco, N = 250) and Asia (Nepal and Turkey, N = 483). The similarity between the species spontaneously listed by non-French (N = 1,840) *versus* French children (N = 647) was very high (Morisita-Horn similarity index = 0.751). Whatever the country, children essentially refer to a few iconic mammals, suggesting a strong uniform influence of the media. We also performed internet surveys (as presented above) using English, Spanish or Italian. The main outcome is that whatever the language used, the same few iconic species occupy most of the space (comparing non-French *versus* French surveys; Morisita-Horn similarity index = 0.905). In fact, the similarity between the lists of species that dominated the responses was even stronger through the media comparison than with the children; a result somehow expected given the worldwide homogeneity of the messages about animal conservation (big cats, bears, dolphins and whale plus a few other icons clearly dominate). Below we examine in more details the relationship between the media and children, along with potential consequences in terms of environmental education.

Both the internet and the schoolchildren surveys enabled us to identify ∼150 “priority protection species” (Figure 1). A superficial examination of this result could be interpreted as an encouraging message in terms of conservation of biodiversity. A far more pessimistic view is conceivable, however. Pooling schoolchildren and the internet, only 256 different “priority protection species” were counted, representing less than 3% of all threatened animal species listed by the IUCN, and 4% of all threatened vertebrates. Importantly, both values strongly underestimate the actual numbers of threatened species (IUCN 2010). Clearly, most of the animal species are neglected due to the preference for very few charismatic icons. This contrasts with the fact that children have tremendous capacity for learning about creature identity and characteristics. Young children are able to recognize every single specimen of the 493 Pokémon “species” (e.g., a value three times greater than our number of “priority protection species”), but they face great difficulties when asked to recognize common animal species [18]. Although, our estimate of “priority protection species” richness provided by the media was limited to the internet and by the techniques we employed for data collection, our results suggest that the major media focused information on a few iconic and exotic species. This is particularly problematic, because the internet is currently one of the main sources of information [25]–[28], [30], [31].

Various media sources have a strong impact on the development of human's attitudes toward wildlife [34], [50]–[51]. Accordingly, we detected a strong similarity in the patterns of “priority protection species” obtained from the internet search and from the questionnaires administered to the children. The internet (or other media such as television, assuming that they also broadcast narrowly-focused messages on a few iconic species) appears to be one of the main channels used by children to gather information on biodiversity conservation issues (either directly, or indirectly through parents, teachers, educators, etc.). The media are focused on a few charismatic and flagship species [35] which are consequently predominant among the species cited by school children (e.g., the giant panda or the polar bear, see results). Because of their natural attractiveness, flagship species are used as conservation tools to raise conservation awareness and funding. However, it has been shown that focusing too heavily on these species detracts conservation efforts from other species and projects [35], [52]–[53]. In fact, a study even suggested that the information provided by the media has currently no direct value for the conservation and protection of large groups of charismatic fauna [54]. We do not adopt such a pessimistic view and we nonetheless consider that the media have the virtue to raise ecological awareness, and hence that they are useful. But we also emphasize that there is a taxonomic bias in orienting the general public to protect not-threatened species to the detriment of general biodiversity (e.g., domestic cats and dogs were among the most cited “priority protection species”, unpublished data). Such bias unfortunately extends to professional researchers and official conservation policies [55], [56].

**Figure 3:**
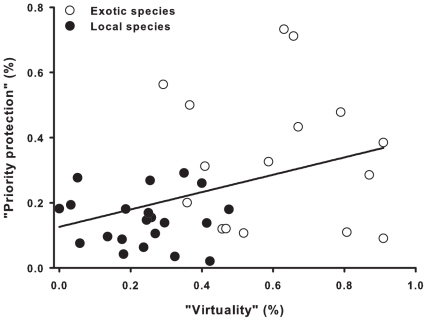
Relationship between “virtuality” and “priority protection”. “Virtuality” is given as a proportion, from 0 for species for which live specimens have already been seen/observed by all of the interviewed schoolchildren, to 1 for species that have never been seen/observed. “Priority protection” is given as a proportion, from 0 for the species that are not declared as “priority protected” by schoolchildren to 1 for the species rated as priority protection by all the interviewed schoolchildren. Exotic and local species are represented by open and filled circles respectively. The equation for the regression line is y = 0.126+0.266x (F_1,36_ = 5.52, p = 0.02).

Disregarding the potential negative effects of the media focusing too narrowly on a very few species, our most worrying result is the meagerness of the knowledge of the children about common local species. Responses to the closed–ended items in the questionnaire showed that children actually had the opportunity to observe common local animal species (75% of the local species presented on the color plates, Figure 2). However, exotic species were more easily identified than local ones. For instance, the toucan (exotic for French children) was recognized by 41% of the children, whereas the European blackbird (a very common and conspicuous species in Western Europe, both in country and urbanized areas) was recognized by only 21% of the children, and some common invertebrates (e.g., house centipedes) or amphibians (e.g., newts) were virtually never identified. Clearly, knowledge of local animals is skeletal [19], [57]. This result supports the existence of a critical and deleterious disconnection between people and their local environment; a fact documented by other researchers [29]. There are two concerns associated with this issue. Firstly, people care only about what they know [18], [32]. Secondly, and probably more importantly, schoolchildren may well be more prone to protect exotic and hence somehow virtual Biodiversity rather than their own local species (see Figure 3 for the relationship between the level of “priority protection” and the level of “virtuality” of the species displayed on the color plates). Such disconnection can explain paradoxical attitudes and behavior, such as the abuses of pesticides in the gardens of people that nonetheless consider themselves as concerned by the decline of tigers in the wild. A widespread referral to virtual nature or virtual biodiversity, combined with the extinction of vicarious experiences tends to devalue local environment by substituting essential direct and emotional experiences of local natural areas by virtual ones [40], [42].

The poor knowledge and low consideration to protect local species as a priority that we detected is problematic and most worrying. Indeed, all studies on these issues converge on the fact that to be effective, conservation awareness must be heavily based on local biodiversity, on the species from our own backyards and gardens [32], [58], [59]. Knowledge of the most common local organisms is crucial: in practice, most individuals have far greater opportunities to efficiently protect local biodiversity rather than to protect exotic species (e.g., signing a petition). In this respect, both the media and environmental education (notably at school) have key roles to play. Schools are crucial for the creation of positive attitudes toward global biodiversity and are even expected to compensate for parents' lack in this regard [60]. There is, however, a strong disparity between what should be and what is done [61]. Environmental education mediated by local experiences is declared as a key component in academic programs, but practical actions are usually not encouraged [4], [62]. Very little time (if any) is spent on direct observations of plants and animals and field experiences have declined considerably over time [29], [38], [54], [63]. This is particularly regrettable because even school playgrounds, and not necessary wild forests, are extremely valuable settings for investigations in nature both in urban and rural areas [19]. The use of such anthropized sites engage little or no travel costs, little time; and could be used in long-term projects (e.g., a simple monitoring of snail populations would be costless, fascinating and rewarding for the schoolchildren). Learning about animals in their natural habitats may result in higher knowledge scores than would any lessons in the classroom [19]. Exploring biodiversity in the natural surroundings of a school can target young people that are not traditionally reached by science outreach or biodiversity-related volunteering programs [64]. Such type of projects also allows citizens to be involved in research and may give them opportunities to be engaged in a conservation career. The rarity of educational programs based on both field experience and non-iconic animals is particularly unfortunate considering successful initiatives such as the Iimbovane Outreach Project in South Africa that explores biodiversity in school grounds and surrounding natural areas based on ants' ecology and diversity as a mean to connect children to their environment (http://academic.sun.ac.za/Iimbovane/index.htm).

Both the media and schools have the responsibility to engage children in developing favorable attitudes toward biodiversity. In the current context of strong biodiversity decline, the successful awareness raising among people and children with a few charismatic animals, although important, is clearly insufficient. Natural attractiveness of children towards animals should not focus only on few iconic species but must be also directed toward common and local organisms by engaging children with practical experiences with nature. Our study simply adds another call to push the children outside and away from the screens.
